# Supplementary material for: Assessment of the Influence of Size and Concentration on the Ecotoxicity of Microplastics to Microalgae Scenedesmus sp., Bacterium Pseudomonas putida and Yeast Saccharomyces cerevisiae
Source: Polymers (Basel). 2022 Mar 19;14(6):1246. doi: 10.3390/polym14061246 (PMC8952821; doi:10.3390/polym14061246)
Supplement: Supplementary file 1 [file polymers-14-01246-s001.zip › polymers-1597482-supplementary.pdf]

Supplementary Materials

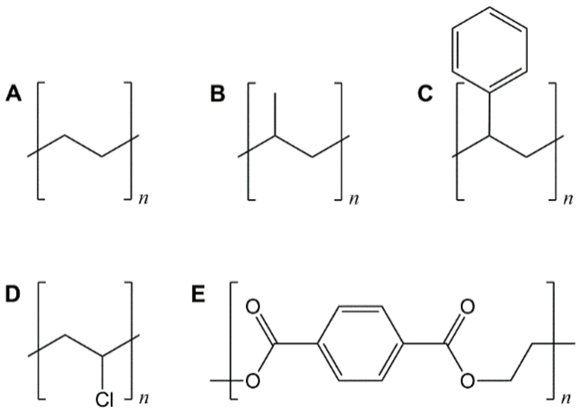

Figure S1. Structures of the applied microplastics: (**A**) polyethylene, (**B**) polypropylene, (**C**) polystyrene, (**D**) polyvinyl chloride, and (**E**) polyethylene terephthalate.

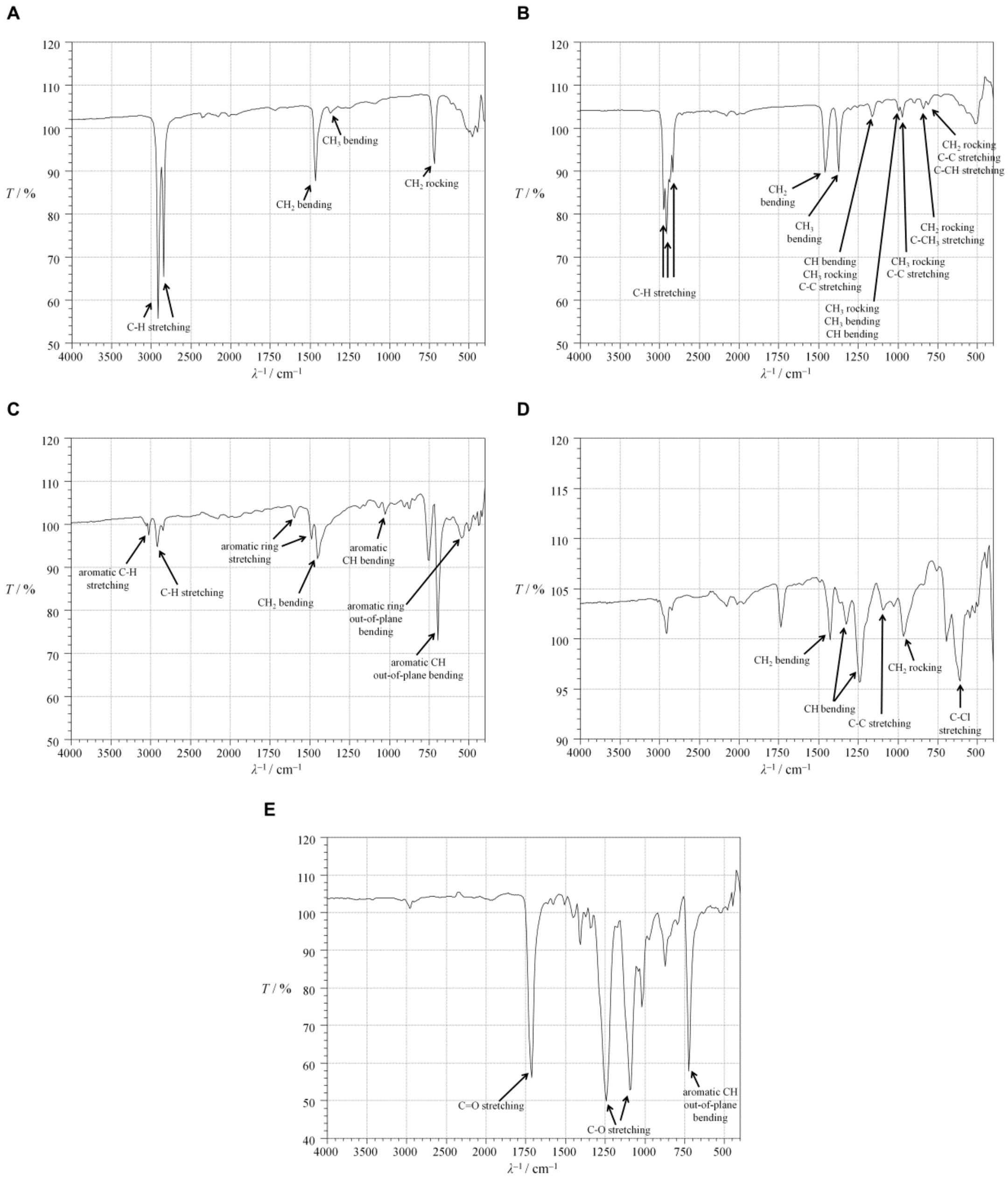

Figure S2. ATR-FTIR spectra of: (**A**) polyethylene, (**B**) polypropylene, (**C**) polystyrene, (**D**) polyvinyl chloride, and (**E**) polyethylene terephthalate.

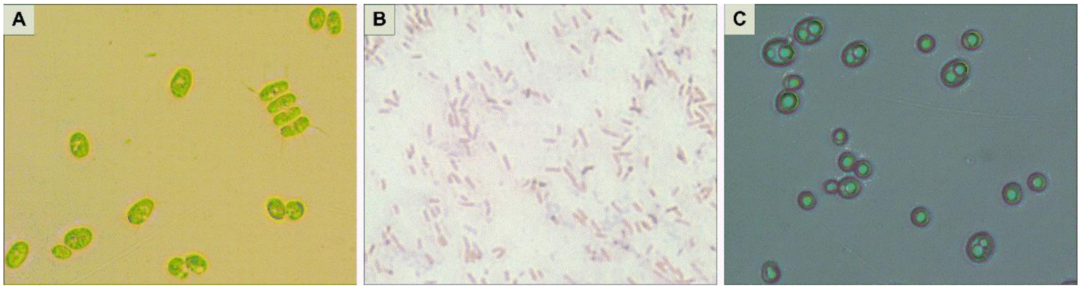

Figure S3. Microscopic images of test organisms used in the research: (**A**) microalgae *Scenedesmus* sp. (magnification 400×), (**B**) bacteria *Pseudomonas putida* (magnification 1000×), and (**C**) yeast *Saccharomyces cerevisiae* (magnification 400×).
